# Delayed diagnosis of post-traumatic C7 vertebra anterior subluxation with an unusual neurological pattern: a case report

**DOI:** 10.1186/1752-1947-7-33

**Published:** 2013-01-31

**Authors:** Maryam Sanaullah, Abdul Sattar Mohammad Hashim, Ayesha Sundus, Sanaullah Bashir, Maheen Rehman

**Affiliations:** 1 Dow Medical College, Dow University of Health Sciences, Baba-e-Urdu Road, Karachi, 74200, Pakistan; 2 Department of Neurosurgery, Jinnah Post Graduate medical Center, Rafiquee Shaheed Road, Karachi, 75510, Pakistan; 3 Civil Hospital Karachi, Dow University of Health Sciences, Baba-e-Urdu Road, Karachi, 74200, Pakistan

**Keywords:** Anterior subluxation, Spine, C7 vertebrae, Trauma

## Abstract

**Introduction:**

Post-traumatic subluxations are potentially devastating injuries to the axial skeleton. Of utmost priority are an expedient and timely diagnosis and realignment because of its association with spinal cord and nerve root trauma, which lead to progressive deleterious neurological deficits. A good radiological study of the occipitocervical joint and first thoracic vertebra is key to a successful early diagnosis. However, cases might still fail to be diagnosed, leading to trouble. A case of post-traumatic subluxation at the C7 vertebral level with an unusual neurological pattern is presented here.

**Case presentation:**

A 35-year-old farmer from the Sindh province of Pakistan presented to our neurology department after a fall 2 months earlier and complained of lower limb pain and difficulty in walking. He had numbness in both of his lower limbs up to his umbilical region, with sparing of bladder function along with intact strength in the upper extremities bilaterally.

**Conclusions:**

Our case highlights the unusual sparing of upper limbs and intact urinary continence with severe lower limb deficits in a 70% subluxation. Our case is unusual because highly detrimental effects such as quadriplegia are expected with such extreme subluxation, but our patient presented with only lower limb deficits. This case serves as a reminder to emergency medicine doctors, spine surgeons, and even radiologists (a) to evaluate spine injuries by using computed tomography in trauma patients to identify artifact around a suspected injury and (b) to be mindful of negative conventional radiographs.

## Introduction

Subluxation is defined as a partial dislocation, which is any pathological situation in which there is not a normal physiological juxtaposition of the articular surfaces of the joint [1]. A traumatic accident can lead to neglected spinal injuries if the patient experiences multiple injuries. About 6000 to 10,000 people in the US sustain spinal cord injuries each year. About 55% to 75% of these injuries are caused by auto accidents and falls, and the remainder result from collisions or falls during sports and other recreational activities [2].

Neglected spinal injuries are defined as injuries not treated in a timely fashion and found late when options are limited [3]. They can go undiagnosed if radiographs are not available or are misread, if a radiograph fails to demonstrate a lesion properly, or if the patient has had multiple traumas or is unconscious and intoxicated. Such injuries are four and a half times more common in the cervical spine than the thoracolumbar spine [3]. Radiological studies provide a key to a good diagnosis. Technical measures should be taken while obtaining X-rays because a slight movement of the shoulders might result in superimposed images. If X-rays are not satisfactory, computed tomography (CT) and magnetic resonance imaging (MRI) are indicated in order to get a complete and unambiguous picture of the situation. We report a case of a delayed diagnosis of a 35-year-old with post-traumatic anterior subluxation of the C7 vertebra. To the best of our knowledge, such severe compression would usually lead to disastrous effects in both upper and lower limbs and in many cases quadriplegia, but our case represents an unusual presentation of symptoms only in the lower limbs.

## Case presentation

A 35-year-old man from the Sindh province of Pakistan presented to our neurology emergency room with numbness in both lower limbs up to his umbilicus and difficulty in walking for the previous 15 days. He had fallen from a tree 2 months earlier and had no bleeding fraction and an unremarkable shoulder X-ray. He experienced mild pain in his right shoulder and neck after the trauma, which was relieved through medication, and was sent home.

A physical examination displayed a good physique and a normal body mass index. There was no visible or palpable stepoff in the spine, and no tenderness was observed around the area of fracture. A detailed neurological examination revealed that his upper limbs were normal, power was 5/5 according to the Medical Research Council (MRC) scale, and all sensations were intact, whereas his lower limbs demonstrated decreased bulk and tone. The power in both of his lower limbs was 4/5 according to the MRC scale, and his left leg showed exaggerated deep tendon reflexes. His left leg exhibited withdrawal on eliciting plantar reflex, whereas his right leg was mute. Superficial abdominal reflexes were absent. In a sensory examination, our patient had diminished vibration sensation in both lower limbs up to his umbilicus. Below his umbilicus, increased pin prick (superficial pain) sensation and hypersensitivity to cold were detected. The results of the rest of the sensory examination were unremarkable. Our patient had no difficulty in breathing and showed normal pulses and vascularity in his arms and legs. Laboratory investigations, performed to exclude infection or metabolic disorders, all had normal results except for mild anemia.

**Figure 1:**
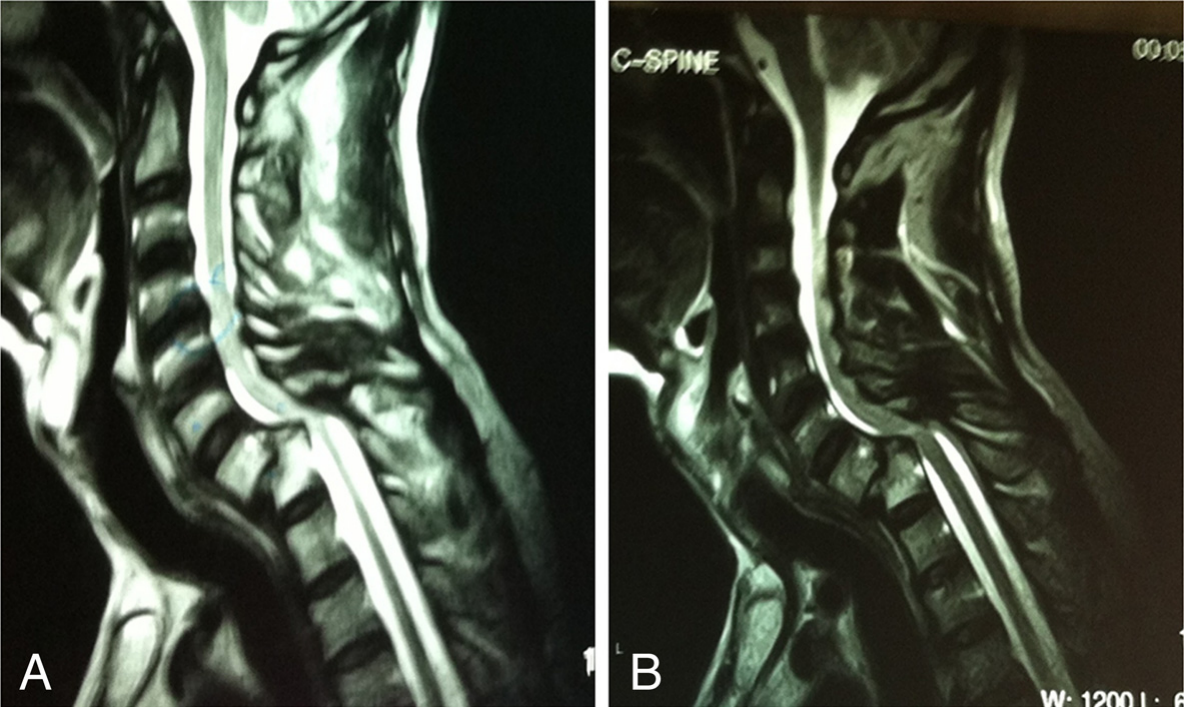
**Computed tomography cervical spine sagittal images.** Computed tomography images with **(A)** and without **(B)** contrast reveal severe cord compression at the level of C7.

**Figure 2:**
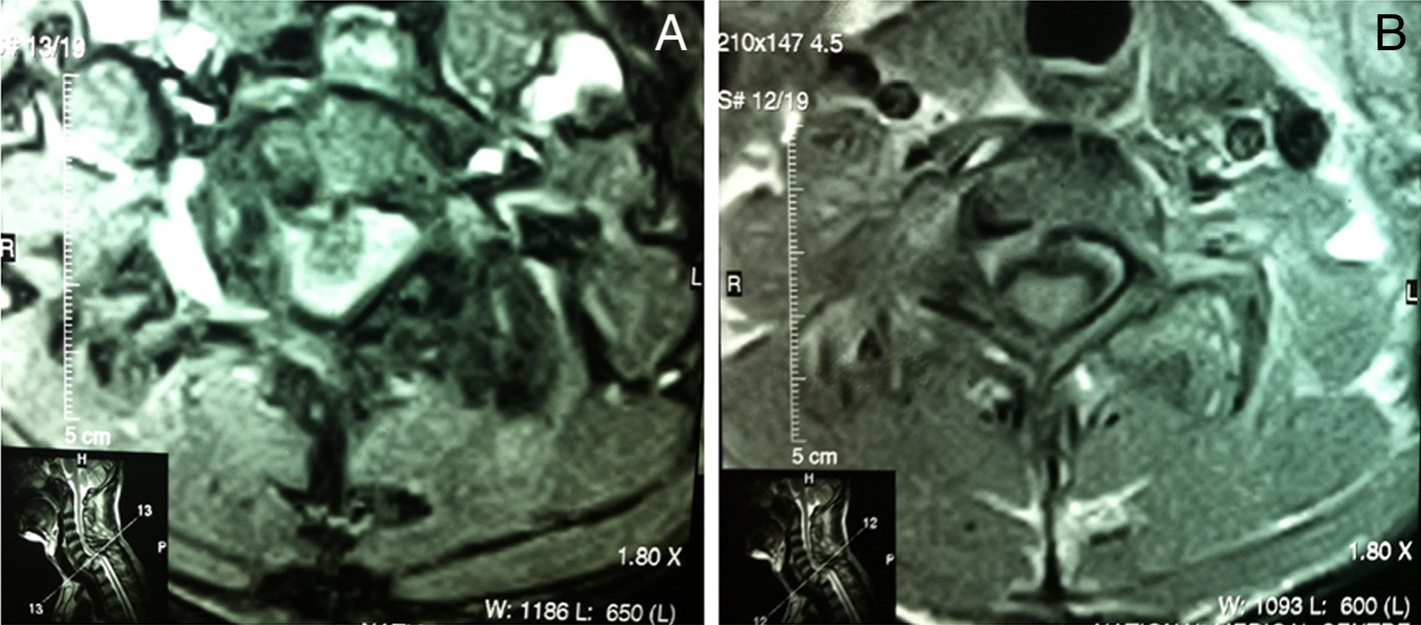
**Computed tomography cervical spine axial images.** Computed tomography images with **(A)** and without **(B)** contrast reveal subluxation of C7 and a major compression of the spinal cord.

A CT scan of the cervical spine showed a fracture of the lamina of C7 bilaterally and a bilateral anterior dislocation of facet joints, resulting in bilateral facet lock, and grade IV antererolisthesis of C7 over T1, causing a marked narrowing of 0.6cm anterio-posteriorly at this level of the spinal canal and a compression over the spinal cord. A fracture of the right inferior articular facet of C7 and an avulsion fracture of the anterior inferior corner of T1 were also noted (Figures 1 and 2).

Our patient’s neck was immobilized by using a cervical collar as part of conservative management. Later, he underwent a T12 anterior cervical corpectomy with fusion and plating while under general anesthesia. A massive abdominal aortic aneurysm was observed. A vertical incision along the anterior border of the left sternocliedomastoid was performed. The T12 vertebra was expanded, and an anterior corpectomy was performed. A graft was taken along the right iliac crest, and fusion was carried out and copper plating was performed to impart immediate stability to allow optimal bone healing and fusion. A drain was placed, and closure layer by layer and aseptic dressing were performed. Post-operative orders were to have nothing by mouth for 6 hours following the procedure.

At the 3-month follow-up, there were no abnormal neurological sequelae, motor power improved, and our patient was able to walk. He improved neurologically and became ambulant without support.

## Discussion

Bilateral facet dislocations at subaxial levels in a flexion-distraction-type trauma are three-column injuries that are very unstable and frequently associated with devastating neurological deficits [4,5]. Our patient was not immobilized when he first presented with pain after his fall, which led to progressive degeneration and gradually increasing subluxation and to unusual severe lower limb symptoms 2 months later. Early recognition and management of cervical spine injuries in the patient with acute trauma are necessary to prevent detrimental neurological outcomes [6].

Since bilateral dislocations of the facets compress the spinal cord and nerve roots, early reduction of the locked facets and decompression are critical in preventing progressive secondary spinal cord injury [5,7]. Our patient had a delayed diagnosis, so the dislocation that initially was not so severe slowly progressed to 70% subluxation at the time he presented with his unusual symptoms. A CT scan was performed at this point rather than initially. CT has been shown to have a sensitivity of 98%, and when CT is combined with the standard three-view series, a sensitivity of 100% has been demonstrated [8].

The patient in this case had a delayed diagnosis because no neck X-ray was taken after his fall and the X-ray of his shoulder was normal. A shoulder X-ray would not have revealed subluxation and hence he presented 2 months later with a secondary problem. His neck pain was milder than the shoulder pain and therefore was not investigated by either X-ray or CT. Trauma patients should therefore undergo a CT scan if they have any complaints of neck pain after trauma, especially after a fall from a great height, as in this situation. The general “rule” is that films are needed when any one of five criteria are involved: neck pain, neck tenderness, the patient is not alert or is under the influence of alcohol or drugs, any neurological abnormality or symptoms, or other distracting injuries [9,10]. Harris [9] states that the incidence of missed cervical spinal cord injuries has more than doubled during the past decade. He sug-gests that the primary physician/trauma surgeon in the emergency department must maintain a high degree of suspicion regarding the possibility of spinal cord injury in all patients with blunt trauma and must be mindful that a “negative” conventional radiographic examination in the presence of an abnormal clinical evaluation requires further diagnostic tests, including CT or MRI.

Neck immobilization should be an important part of conservative management in spinal injuries. If our patient had been provided with a safe and expedient realignment the first time he presented after his fall, the dislocation would not have progressed to the unusual secondary deficits. Complete spine immobilization is mandatory until stabilization is achieved [10,11]. However, prolonged immobilization has its own deleterious effects [10,11]. During cervical spine precautions, patients with obtunded trauma are immobilized and can develop pulmonary complications and deep vein thromboses [12]. Airway management is more difficult in patients with cervical collars and may also lead to skin breakdown and ulceration [13].

Anterior stabilization was performed to provide a firm support and alleviate the compression of the spinal cord. The anterior approach provides direct access to the cord and the compressive elements and has a lower incidence of post-surgical kyphosis [12]. Highly detrimental effects are expected in such a severe compression, but this case presented an unusual sparing of the upper limbs, and the patient was totally continent throughout while his lower limbs displayed significant symptoms secondary to compression. Bilateral facet dislocation of C4 through C7 creates instability and frequently is associated with quadriplegia [14]. The sparing of the upper limbs indicates that, even with 70% subluxation, injury to the cord and nerve roots was incomplete. This could be due to progressive degeneration over the course of 2 months rather than to a single severe traumatic injury to the spine.

## Conclusions

This case serves as a reminder to emergency medicine doctors, spine surgeons, and even radiologists that spine injuries should be evaluated by CT in patients with trauma in order to identify artifact around a suspected injury. If the results seem uncertain, the tests should be repeated or alternative imaging forms should be used to avoid a delayed diagnosis and subsequent harmful effects.

## Consent

Written informed consent was obtained from the patient for publication of this manuscript and accompanying images. A copy of the written consent is available for review by the Editor-in-Chief of this journal.

## Abbreviations

CT: Computed tomography; MRC: Medical Research Council; MRI: Magnetic resonance imaging.

## Competing interests

The authors declare that they have no competing interests.

## Authors’ contributions

MS developed the idea for the case report, wrote part of the report, and reviewed it. ASMH headed the study, is the chair of the department, revised the draft of the case report, and provided a critical review of the study. AS and MR helped to write the Discussion section. SB helped with the examination of the patient and wrote the Case presentation section. All authors read and approved the final manuscript.
